# Current Status of the Open Abdomen Treatment for Intra-Abdominal Infection

**DOI:** 10.1155/2013/532013

**Published:** 2013-10-02

**Authors:** Yujie Yuan, Jianan Ren, Yulong He

**Affiliations:** ^1^Department of Gastrointestinal and Pancreatic Surgery, The First Affiliated Hospital of Sun Yat-Sen University, 58 2nd Zhongshan Road, Guangzhou, Guangdong 510080, China; ^2^Department of Surgery, Jinling Hospital, Medical School of Nanjing University, 305 East Zhongshan Road, Nanjing, Jiangsu 210002, China

## Abstract

The open abdomen has become an important approach for critically ill patients who require emergent abdominal surgical interventions. This treatment, originating from the concept of damage control surgery, was first applied in severe traumatic patients. The ultimate goal is to achieve formal abdominal fascial closure by several attempts and adjuvant therapies (fluid management, nutritional support, skin grafting, etc.). Up to the present, open abdomen therapy becomes matured and is multistage-approached in the management of patients with severe trauma. However, its application in patients with intra-abdominal infection still presents great challenges due to critical complications and poor clinical outcomes. This review focuses on the specific use of the open abdomen in such populations and detailedly introduces current concerns and advanced progress about this therapy.

## 1. Basic Conception of the Open Abdomen Treatment

The open abdomen (OA) treatment, defined as leaving the fascial edges of abdomen unapproximated intentionally, is one of the greatest surgical advances in the twentieth century [1]. This treatment was stemmed from the concept of damage control surgery (DCS), which was first coined by Rotondo et al. in 1993 [2]. Nowadays, the OA procedure has been widely applied and improved clinical outcomes since it was first popularized in the mid-1990s [3, 4].

 To date, an increasing intra-abdominal pressure (IAP) >15 mmHg with the onset of newly formed organ failure is an indication for a prompt OA procedure [5]. Actually, the indications of open abdomen have extended to several recognized conditions (Table 1). Of note, the OA therapy can be indicated in cases with intra-abdominal infection (IAI) or sepsis when a single laparotomy failed to control the source of infection or the risk of organ dysfunction increased after effective drainage and debridement. This therapy has been described in several types of IAI, such as purulent, fecal, and secondary peritonitis [6–10]. The mortality rate in those patients could decrease by almost 50% after an effective OA approach [11]. However, the use of open abdomen in IAI patients still presents great challenges due to critical complications and poor clinical outcomes. Hence, the review mainly focuses on the current problems and advanced progresses of OA treatment for patients with IAI. 

 Nonclosure of abdominal fascia with an open abdomen, followed by a temporary abdominal closure (TAC), has become a major advance in treatment of IAI [5, 12]. This staged approach covers many clinical benefits, especially in preventing the development of secondary intra-abdominal hypertension (IAH) or subsequent abdominal compartment syndrome (ACS) [12], while this aggressive procedure also produces several problems that challenge both surgeon and nurse and has become a controversial strategy in treatment of IAI or sepsis patients. 

## 2. Pathophysiology of the Open Abdomen

**Figure 1:**
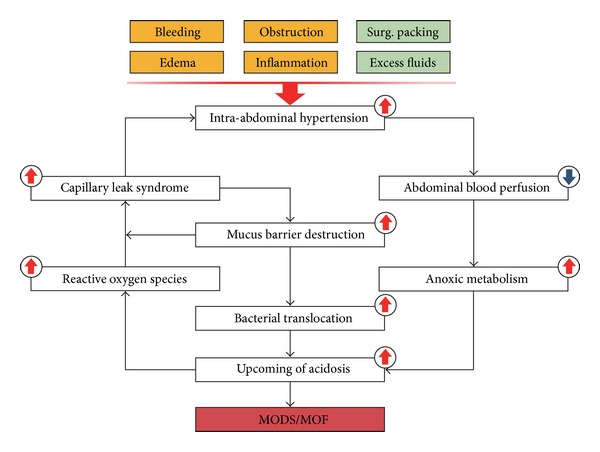
The vicious cycle of persistent intra-abdominal hypertension in patients with IAI. In clinical practice, iatrogenic measures such as surgical packing and fluid treatment would result in the development of intra-abdominal hypertension. All clinical conditions listed here should be paid close attention. This cycle provides sufficient information for the poor outcomes of IAH once the persistent hypertension is not well controlled.

A persistent IAH, defined as an IAP over 12 mmHg, is often associated with a poor outcome due to its induced vicious cycle (Figure 1). A direct effect of open abdomen on peritoneal cavity is a rapid decline in IAP, often decreased by 40–75% [13–15]. Besides, physiologic homeostasis, such as fluid and electrolyte balance, can be temporarily recovered back to normal state soon after an open abdomen [2]. After 48 hours, a secondary materialization of fibrin within the exudate forms a gelatinous mass in which the omentum and bowels can be loosely fixed [16, 17]. During the next 4-5 days, this loose coagulum would be replaced by the increased adhesions, since polymerization of fibrin occurs and collagen is laid down [18]. A so-called frozen abdomen mainly develops within this period, which makes a primary fascial closure almost impossible [19]. Beyond 10 days of an OA procedure, the wound area would be covered with matured granulation tissues and sufficient microvascular circulation, which indicates a process of fibrin deposition and collagenization [20].

Specifically in severe IAI, the outbreak of the disease is usually 6–12 h before a surgical intervention, and a considerable accumulation of inflammatory cytokines (TNF-*α*, IL-1, IL-6, IL-10, etc.) around infected areas has already occurred, followed by a systemic inflammatory response cascade. These serial responses would cause microvascular dysfunction, massive visceral edema, and IAH, which require a sufficient source control to terminate. A proper OA procedure can provide repeated access to the peritoneal cavity for required debridement of devitalized tissue, peritoneal effluent, and surgical drainage [20, 21]. Besides, a newly formed retroperitoneal hematoma from IAI or some iatrogenic interventions, such as inadequate intra-abdominal packing and overload fluid resuscitation, would possibly increase the IAP and cause the development of ACS. Beyond that, the persistent IAP from severe IAI might cause abdominal wall ischemia and abdominal fascia necrosis, which eventually result in abdominal wall rupture with subsequent development of huge ventral hernia. Therefore, a prompt open abdomen must be considered to achieve source control and intra-abdominal decompression.

 Additionally, the OA procedure was reported to promote the recovery of intestinal mucosal barrier from hypertension-induced mechanical damages [22]. Such protective role for mucosal barrier would further reduce the risk of bacterial translocation and subsequent MODS or MOF [23]. 

## 3. Fundamental Steps of the Open Abdomen

The OA treatment generally consists of three sequential steps: a timely laparostomy, a TAC procedure, and a definitive abdominal closure [24, 25]. As for IAI or sepsis, the major challenge of OA management is to control septic peritonitis and intra-abdominal fluid accumulation, while preserving a temporary abdominal closure [26]. Compared with traumatic patients, the last step in septic patients often delayed approximately 4–6 months for dealing with infection, fistulas, bleeding, malnutrition, or other severe complications. Before that, a frozen abdomen was unavoidable and early fascial closure was almost impossible to perform practically [27]. Additionally, septic population was usually order with more comorbidities than traumatic population, which directly increased the risk of complications after an open abdomen. Therefore, primary closure rate in sepsis patients is substantially lower than that in trauma patients [28]. Many infected patients with an open abdomen would develop large and debilitating hernias of abdominal wall, which require additional operation to repair at a later stage [29]. 

## 4. The Open Abdomen in Treatment of Intra-Abdominal Infection

### 4.1. Complications and Outcomes after an Open Abdomen

Although the open abdomen has addressed several lethal problems related to IAI situation, this technique is still correlated with many complications (Table 2). Among those, gastrointestinal fistula is the most serious and challenging for both surgeon and nurse. This specific complication, particularly enteroatmospheric fistula, is hard to prevent, with the overall incidence approximately 5–25% [30–32]. It is almost impossible to achieve a spontaneous closure once the fistula is established. In addition, the surgical intervention is commonly delayed for 3–6 months when the fistula output is limited. These secondary fistulas could corrode the wound areas by those spilled bowel contents, which would further result in local infection, surgical site infection, intra-abdominal abscess, or systemic infection with the potential for electrolyte imbalance [33]. 

As compared with traumatic patients, patients with IAI or sepsis almost had worse outcomes after an open abdomen, such as declined survival rate, increased secondary fistulation, and relatively delayed primary closure rate [34]. Those patients had a high risk of multiple organ dysfunction syndrome or failure (30–40%), intra-abdominal abscess (83%), and ventral hernia (around 25%) [35]. Moreover, clinical outcomes of infected OA became much poorer compared with clean or contaminated OA only, which included increased transfusion requirements, burdened the utilization of health resources, and increased infectious complications, malnutrition due to significant fluid, electrolyte, and protein losses.

### 4.2. Controversies on the Open Abdomen—Early versus Delayed Fascial Closure; TAC Selection

Generally, definitive fascial closure is recognized as the ultimate goal of the OA treatment. In clinical practice, the fascial closure can be achieved with an early fashion or a delayed procedure during the first treatment process. Unfortunately, there is still no consensus about which fashion should have priority in use after a successful open abdomen. Early fascial closure, commonly performed within 9 days of initial laparostomy, usually needs strict monitor of the status of incremental fascial closure to assess the possibility of a definitive operation. This early fascia-to-fascia closure could not be successful if early surgical source control failed [36]. Pliakos et al. indicated that sequential fascial closure could immediately begin once abdominal sepsis is well controlled and often achieved better outcomes than a single use of the vacuum associated closure (VAC) device [37]. 

Nonetheless, patients with IAI or abdominal sepsis are less likely to achieve an early fascial closure [27]. For such populations, the failure attempt was mainly attributed to a delayed wound healing process due to the persistent stimulation of inflammatory mediators. Delayed fascial closure, defined as fascial abdominal closure over 9 days after initial OA procedure, is often performed through a form of planned ventral hernia repair [26]. Besides the primary disease, massive transfusion (also known as overload fluid resuscitation), early presence of complications during hospitalization and nonfascial traction technique were also attributed to the postponed closure [38, 39]. Generally, a definitive fascial closure would be performed at about 6–12 months after an open abdomen. However, many attempts, aiming to achieve a safely early fascial closure in such infected open abdomen, have indicated the possibility of fast recovery from this complicated situation in the future [40].

Although numerous TAC techniques have been applied in the management of infected open abdomen, many of those modalities are not primarily intended to close the infected abdomen, such as skin only, meshes, or zipper. Because most of IAI patients had the fourth type of OA, also known as frozen abdomen with adherent bowel, they could not receive a definitive operation to permanently close the infected abdomen in early stage [41]. Negative pressure applications, typically VAC device, were reported to have superiority over other TAC techniques in reducing fascial tension and abdominal wall retraction [4]. A recent systematic review indicated that the use of Wittmann patch or VAC earned much higher fascial closure rate than other TAC techniques [42]. However, the use of negative pressure devices was connected with increased risk of secondary fistula and delayed fascial closure rate [31, 43]. Recently, some modified VAC methods were innovated to solve those problems and confirmed their effect and safety in clinical practice [35, 44–46].

### 4.3. Advances in the Use of Open Abdomen in Intra-Abdominal Infection

Although the optimal timing to perform a definitive closure remains controversial, a general principle of considering this operation has been made during the last decades. This principle includes the following: (1) IAP is less than 15–20 mmHg when fasciae can be approximated temporarily. (2) No bowel leak is detected, or a low-volume output of bowel fluids is realized. (3) Abdominal wall integrity can be restored based on systematic preoperative evaluation. (4) No wound-healing problems can be anticipated. (5) Infection source is totally eliminated and debridement of peritoneal cavity is sufficient. (6) Nutritional status and pulmonary function are satisfying to tolerate the stress of operation. 

Once again, as for an infected open abdomen, VAC system is strongly advocated thanks to its best overall clinical outcomes [34]. Compared with other negative pressure applications, VAC system could effectively prevent adherence of the viscera to the abdominal wall, increase the primary fascial closure rate, and have a nice performance in source control in septic patients. Importantly, this technique was cost effective and simple to perform in any levels of hospitals [47–49]. The newly innovative VAC system (ABThera) has the additional feature of inner sponge extensions that extend to the ends of the plastic sheet to facilitate more effective drainage of peritoneal effluent [4, 50]. 

 Besides the advanced progresses in the OA management, the control of OA-associated complications has been further improved. Enteroatmospheric fistula, one of the most challenging issues from infected open abdomen, is almost impossible to be cured without an operation, as proper soft tissues are not available to cover the fragile bowel wall. As a result, prevention remains the most crucial management principle. Whenever possible, biocompatible materials were preferred as the mediators separating exposed viscera from the ambient atmosphere. In addition, routine wound care should be performed by experienced surgeons or enterostomal nursing specialists [51]. Once the fistula was established, aggressive nutritional support and meticulous wound care should be initiated as soon as possible. The VAC system was reported to be effective in the control of fistula effluent with eventual healing of the fistula [5]. Recently, we found that the “fistula patch,” a hand-made silica lamellar, was helpful for the management of enteroatmospheric fistula. This homemade material could bring together the benefits of avoiding the loss of bowel fluids, simplifying wound care, ceasing tissue destruction, and supporting nutrients delivery [52]. 

 The use of enteral nutrition (EN) for patients with an open abdomen remains hesitancy, since EN is always twisted with issues of enteral access, concerns of bowel edema, or dilemma of intestinal motility and function. A recent multi-institutional study demonstrated that EN after an open abdomen was associated with increased fascial closure rates, reduced complications rates, and improved mortality [53]. Besides, early EN could now be delivered successfully via various approaches in patients with IAI, with reduced fistula formation and improved mortality rates [54, 55]. This early initiation of enteral feeding was reported to be an independent predictor of successful fascial closure in OA patients complicated with intestinal fistula [45]. However, prudent concerns must be paid when placing a nasogastric or postpyloric feeding tube to reduce the incidence of anastomotic leaks or fistulae. Those unnecessary complications may compromise future closure approaches and need to be well controlled during the management of infected open abdomen. 

## 5. Expectation of the Open Abdomen Treatment for Intra-Abdominal Infection

The major challenge in treatment of an infected open abdomen is to control septic peritonitis and intra-abdominal fluid secretion and to facilitate repeated abdominal exploration, while preserving the peritoneal fascia for delayed primary closure [26]. Before that, a decision to leave the abdomen open must be made by a surgeon. However, there are no definite criteria to guide the surgeon in decision-making for septic patients at present. More work is required to better define appropriate indications for a proper open abdomen procedure. No matter whether early or delayed fascial abdominal closure was performed, the improving successful closure rate was essential for treatment of the infected open abdomen. Additionally, various techniques, such as VAC plus specific mesh, biocompatible hydrogels and other TAC systems (e.g., abdominal reapproximation anchor system), should be tried to prevent potential complications associated with OA therapy. Meanwhile, some biomarkers with predictive values for upcoming complications, such as PCT, C3, CRP, and TNF-*α*, should be more emphasized in future clinical practice. Since the use of OA therapy in patients with IAI is quite challenging at present, a large amount of clinical studies, with large sample size and randomized control in design, are required for further evaluating its role in this specific area. 

## 6. Summary

In conclusion, the open abdomen is one of the greatest surgical advances in recent decades and earns enormous popularity in the daily management of critical or infected patients. This multistage surgical treatment brings about huge benefits to such populations by avoiding a series of problems from abdominal closure under extensive tension, facilitating damage-control procedures, reducing the risk of intra-abdominal hypertension, and contributing to the early recognition of intra-abdominal catastrophes. However, the use of open abdomen in patients with intra-abdominal infection also brings on many challenges beyond those that might be expected from the primary illness. The appropriate management relies principally on recovering normal physiology and nutritional status, protecting skin and fascia, and preventing upcoming septic shock or multiple organ failures. With experienced and careful management, those challenges could be finely met and turned to achievements. Any innovative techniques, designed to improve fascial closure rate and clinical outcomes, should be encouraged to apply in an infected open abdomen. It is the responsibility of the clinician to apply management principles judiciously to obtain the most benefits from the open abdomen. Although the open abdomen treatment for intra-abdominal infection does seem to work, prospective randomized controlled trials are warranted to further clarify its role in such specific populations. 

## Figures and Tables

**Table 1:** The recognized indications for the open abdomen treatment.

Indication	Specific situation vignettes
IAI	(1) Source control unsatisfied; (2) SIRS or sepsis predicted; (3) hypovolemic shock resulted from complicated fluid loss or hemorrhage unavoidable; (4) immunocompromised status presented.

DCS for severe trauma	(1) Death triangle (hypothermia <35°C, severe acidosis with base deficit >15 mmol/L, and coagulopathy) emerged; (2) the abdomen cannot be closed primarily due to extensive abdominal wall defection; (3) life-threatening intra-abdominal bleeding suspected or confirmed; (4) interventional therapy for hemostasis failed.

Persistent IAH/ACS	(1) IAP by bladder pressure measurements >20 mmHg more than 48 h; (2) sustained IAP >20 mmHg (with/without an abdominal perfusion pressure <60 mmHg) and at least one organ dysfunction present, in particular for kidney dysfunction. (3) Pulmonary and cardiac function declined significantly; (4) other decompression measures (percutaneous drainage, diuresis, etc.) unsatisfied.

Acute mesenteric ischemia	(1) The need for a mandatory “second look” to evaluate bowel viability and resect additional ischemic bowel segments if necessary; (2) persistent IAH developed, complying with ileus or intestinal necrosis.

Necrotizing infection of the abdominal wall	(1) The infection mainly originated from the endogenous microflora, frequently associated with complications of initial laparotomy; (2) bacterial translocation can be predicted through clinical indexes; (3) necrotizing tissues cannot be repaired from conventional therapies; (4) complicated compartment syndrome occurred.

IAI: intra-abdominal infection; IAH: intra-abdominal hypertension; IAP: intra-abdominal pressure; ACS: abdominal compartment syndrome; SIRS: systematic inflammatory reaction syndrome.

**Table 2:** Open-abdomen-associated complications in patients with intra-abdominal infection.

Complication	Possible reasons
Enteroatmospheric fistulae	(1) The bowel is exposed into air and allowed to desiccate; (2) “biomaterial adherence” to the bowel would lead to transmural changes of the bowel wall; (3) bowels became edema and vulnerable to bacteria invasion due to the capillary leak syndrome; (4) persistent negative nitrogen balance complicated from IAI; (5) decreased intestinal microvessel circulation from IAH or surgical packing; (6) delayed perforation due to operation associated injuries.

Fluid, protein, and heat loss	(1) A large, moist surface area of the intestine is exposed and could suffer huge evaporative water losses, further deteriorated if enteroatmospheric fistula occurred; (2) the increased metabolic demands during IAI, combined with the loss of bowel motility; (3) relatively poor nutrition status and rapid accumulation of third space fluid.

Bleeding/hemorrhage	(1) Given the rich blood supply of bowels and splanchnic organs, the risk of bleeding is significantly increased, especially when inflamed or traumatized bowel wall is exposed to air; (2) the infected patients with an open abdomen often have an associated coagulopathy from hypothermia, acidosis, hypotension, dilution of blood volume, and uncontrolled exhaustion of clotting factors; (3) extensive complement activation or complement depletion disrupts the coagulation system.

Postoperative ileus	(1) Massive electrolyte loss from the exposed wound areas after an open abdomen, in particular for potassium and magnesium; (2) postoperative adhesion often occurred after the initial operation.

Abdominal wall hernia	(1) Extensive abdominal wall defect cannot be repaired with skin-only closure; (2) planned reconstruction surgery is required due to a wide resection of abdominal fascia in initial OA procedure.

Bacterial translocation/ sepsis/MODS/MOF	Mucous damages from the capillary leak syndrome and vicious cycle related to infected open abdomen (Figure 1).

SSI/VAP/ARDS/UTI	Declined immune function because of sustained infection status; iatrogenic infection.

Intra-abdominal abscess	Concealed infection source or secondary perforation fixed by greater omentum.

DVT/PE	Uncommon.

SSI: surgical site infection; VAP: ventilator-associated pneumonias; DVT: deep vein thrombosis; PE: pulmonary embolism; ARDS: acute respiratory distress syndrome; UTI: urinary tract infection.
